# Differential diagnostic value of CD5 and CD117 expression in thoracic tumors: A large scale study of 1465 non-small cell lung cancer cases

**DOI:** 10.1186/s13000-015-0441-7

**Published:** 2015-12-08

**Authors:** Mark Kriegsmann, Thomas Muley, Alexander Harms, Luca Tavernar, Torsten Goldmann, Hendrik Dienemann, Esther Herpel, Arne Warth

**Affiliations:** Institute of Pathology, Heidelberg University, Im Neuenheimer Feld 224, Heidelberg, Germany; Translational Research Unit, Thoraxklinik at Heidelberg University, Heidelberg, Germany; Clinical and Experimental Pathology, Research Center Borstel, Borstel, Germany; Department of Thoracic Surgery, Thoraxklinik at Heidelberg University, Heidelberg, Germany; Translational Lung Research Center (TLRC), Member of the German Center for Lung Research, Heidelberg, Germany; Airway Research Center North (ARCN), Member of the German Center for Lung Research, Borstel, Germany

**Keywords:** NSCLC, Thymic carcinoma, CD5, CD117, Mediastinal mass

## Abstract

**Background:**

Thoracic pathologists are frequently faced with tissue specimens from intrathoracic/mediastinal tumors. Specifically the differentiation between thymic and pulmonary squamous cell carcinomas (SqCC) can be challenging. In order to clarify the differential diagnostic value of CD5 and CD117 in this setting, we performed a large scale expression study of both markers in 1465 non-small cell lung cancer (NSCLC) cases.

**Methods:**

Tissue microarrays of formalin-fixed paraffin-embedded resection specimens of 1465 NSCLC were stained with antibodies against CD117 and CD5. Positivity of both markers was correlated with clinicopathological variables.

**Results:**

CD117 was positive in 145 out of 1457 evaluable cases (9.9 %) and CD5 was positive in 133 out of 1427 evaluable cases (9.3 %). 28 cases (1.9 %) showed coexpression of CD117 and CD5. Among the 145 cases that were positive for CD117, 97 (66.8 %) were adenocarcinomas (ADC), 34 (23.4 %) were SqCC, 5 (3.4 %) were adenosquamous carcinomas (ADSqCC), 8 (5.5 %) were large cell carcinomas (LC), and one (0.6 %) was a pleomorphic carcinoma (PC). In the CD5 positive group consisting of 133 cases, 123 (92.4 %) were ADC, 0 (0 %) were SqCC, 4 (3.0 %) were ADSqCC, 3 (2.2 %) LC and 3 (2.2 %) were PC. None of the 586 SqCC showed expression of CD5. No association of CD117- or CD5 positivity to patients’ age, pathological stages or to T-, N-, or M- categories was observed.

**Conclusions:**

A substantial subset of NSCLC exhibit positivity of CD117 and CD5. Since CD5 expression was not observed in pulmonary SqCC, but is expressed in the majority of thymic squamous cell carcinomas, the application of this immunomarker is a valuable tool in the differential diagnosis of thoracic neoplasms.

**Electronic supplementary material:**

The online version of this article (doi:10.1186/s13000-015-0441-7) contains supplementary material, which is available to authorized users.

## Background

Comprehensive morphological and immunohistochemical subtyping of tumors is of growing importance for therapy selection and propelled the concept of a tumor-specific, individualized treatment. For non-small cell lung cancer (NSCLC) the current WHO Classification [1] therefore extended the concept of immunophenotyping from biopsies to resection specimens. However, the vast majority of NSCLC cases remain non-resectable at initial diagnosis where often only small biopsies or even cytology material is available. Thoracic pathologists are frequently faced with tissue specimens from central tumors with mediastinal involvement. In this setting it is particularly challenging to distinguish pulmonary from thymic primaries [2]. With approximately 80 % [3, 4] the most common phenotype of malignant thymic tumors is squamous cell carcinoma; thus, the differentiation of squamous cell carcinoma of the lung (SqCC) is challenging. However, differentiation of both is of high clinical importance since therapies differ substantially [5] and thymic carcinomas are associated with a better prognosis [6]. Since thymic tumors show a high variability with respect to their phenotype [7], but occur with low frequency, the establishment of specific immunomarkers for the differential diagnostic setting requires large scale phenotyping approaches. CD117 [8, 9] and CD5 [10–13] are well-known diagnostic markers for thymic carcinomas and are frequently used to separate thymomas from thymic squamous cell carcinomas. However, data on both markers in NSCLC is limited. In order to clarify the differential diagnostic value of CD117 and CD5 to separate between pulmonary and thymic primaries, we performed a large scale expression study of both markers in 1465 NSCLC and correlated the findings with common clinicopathological variables.

## Methods

### Cohort characteristics and TMA construction

Formalin-fixed paraffin-embedded specimens of NSCLC resected from 2002 to 2010 at the Thoraxklinik at Heidelberg University were extracted from the archive of the Institute of Pathology, Heidelberg University with the support of the tissue bank of the National Center for Tumor Diseases (NCT: project: # 1283). Tissues were used in accordance with the ethical regulations of the NCT tissue bank established by the local ethics committee. A cohort of 1465 patients was identified for TMA construction. Diagnoses were made according to the recommendations of the World Health Organization classification for lung cancer 2015 [1, 14]. Prior to TMA construction, a hematoxylin and eosin (H&E)-stained slide of each block was analyzed in order to select representative tumor-containing regions. A TMA machine (AlphaMetrix Biotech, Rödermark, Germany) was used to extract a tandem 1.0 mm cylindrical core sample from each tissue donor block. The cohort characteristics are summarized in Table 1.

**Table 1:** Basic clinicopathological characteristics of the analyzed NSCLC cohort

Clinicopathological Variables		CD117		*p*-value	CD5		*p*-value	CD117/CD5		*p*-value
		positive	negative		positive	negative		positive	negative	
Mean age at surgery	63 years	63 years	63 years	*p* = 0.79	62 years	63 years	*p* = 0.13	61 years	63 years	*p* = 0.25
Patient gender										
Male	1016 (69.3 %)	89	921	*p* = 0.03	76	910	*p* < 0.01	14	852	*p* < 0.01
Female	448 (30.5 %)	56	390		57	383		10	356	
Histology										
Adenocarcinoma	711 (48.5 %)	97	611	*p* < 0.01	123	564	*p* < 0.01	26	685	*p* < 0.01
Squamous Cell Carcinoma	586 (40.0 %)	34	548		0	575		0	586	
Adenosquamous Carcinoma	55 (3.8 %)	5	49		4	50		1	54	
Large Cell Carcinoma	71 (4.8 %)	8	63		3	67		1	70	
Pleomorphic Carcinoma	42 (2.9 %)	1	41		3	38		0	42	
TNM-Classification										
pT1a	125 (8.5 %)	21	102	*p* = 0.07	16	103	*p* = 0.02	4	121	*p* = 0.10
pT1b	158 (10.8 %)	19	138		20	135		5	153	
pT2a	641 (43.8 %)	62	578		64	554		15	626	
pT2b	245 (16.7 %)	22	221		16	228		2	243	
pT3	248 (16.9 %)	17	230		13	230		1	247	
pT4	48 (3.3 %)	4	43		4	44		1	47	
pN0	755 (51.5 %)	83	667	*p* = 0.16	80	651	*p* = 0.03	16	739	*p* = 0.57
pN1	353 (24.0 %)	27	324		15	330		2	351	
pN2	351 (23.9 %)	35	315		38	309		10	341	
pN3	6 (0.4 %)	0	6		0	4		0	6	
pM0	1426 (97.3 %)	143	1275	*p* = 0.42	129	1261	*p* = 0.77	27	1399	*p* = 0.53
pM1a	6 (0.4 %)	0	6		2	4		1	5	
pM1b	33 (2.3 %)	2	31		2	29		0	33	
Clinical stage										
I A	190 (12.9 %)	25	162	*p* = 0.32	25	156	*p* = 0.05	6	184	*p* = 0.48
IB	344 (23.5 %)	32	312		38	293		7	337	
IIA	277 (18.9 %)	28	248		15	259		3	274	
IIB	165 (11.3 %)	18	145		11	152		1	164	
IIIA	422 (28.8 %)	40	380		39	376		11	411	
IIIB	29 (1.9 %)	0	29		2	25		0	29	
IV	38 (2.6 %)	2	36		3	33		0	38	

### Immunohistochemistry

Immunohistochemistry (IHC) was performed with commercially available antibodies (Additional file 1: Table S1) and was applied according to quality-controlled protocols that are regularly evaluated in round robin trials (http://www.nordiqc.org). In brief, TMA slides were deparaffinized and pretreated with antigen retrieval buffer. Subsequent steps were performed on an immunostaining device (Ventana BenchMark Ultra, Tuscon, USA). Pretreatment and dilution specifications are summarized in Additional file 1: Table S1. Evaluation of IHC was performed blinded to the resection specimen diagnoses, according to a dichotomous scoring scheme [15]. 1 % stained tumor cells were considered to indicate positivity. Each tumor from a patient was represented by two cores on the respective TMA; both cores were analyzed together.

### Statistics

Data analysis was performed using R statistical software (v. 3.2.2) and RStudio (v. 0.98.507). Barplots were created with the ggplot2-package (v. 1.0.1) and dependencies. *T*-test was applied to examine differences in age. Fisher’s exact test of independence or Freeman-Halton test was applied to compare between histotypes, T-, N- and M- categories and clinical stage. A *p*-value <0.01 was considered statistically significant.

## Results

### Patient characteristics

Clinicopathological data of 1465 NSCLC cases (stage IA-IV) were analyzed. The median age at diagnosis was 64 years (range: 30–89 years). The majority of patients were male (69.3 %). 711 patients were diagnosed with adenocarcinoma (ADC; 48.5 %), 586 patients with squamous cell carcinoma (SqCC; 40.0 %), 55 patients with adenosquamous carcinoma (AdSqCC; 3.8 %), 71 patients with large cell carcinoma (LC; 4.8 %) and 42 patients with pleomorphic carcinoma (PC; 2.9 %), respectively. Neither CD117 positivity, nor CD5 positivity correlated to patients’ age (*p* = 0.79 and *p* = 0.13), any T- (*p* = 0.07 and *p* = 0.02), N- (*p* = 0.16 and *p* = 0.03) and M-categories (*p* = 0.42 and *p* = 0.77) or clinical stage (*p* = 0.32 and *p* = 0.05).

### Expression of CD117 and CD5 in NSCLC

In 46 patients (3.1 %) CD117 and/or CD5 could not be evaluated because there were either no vital tumor cells or lost tissue cores during the staining process.

**Fig. 1:**
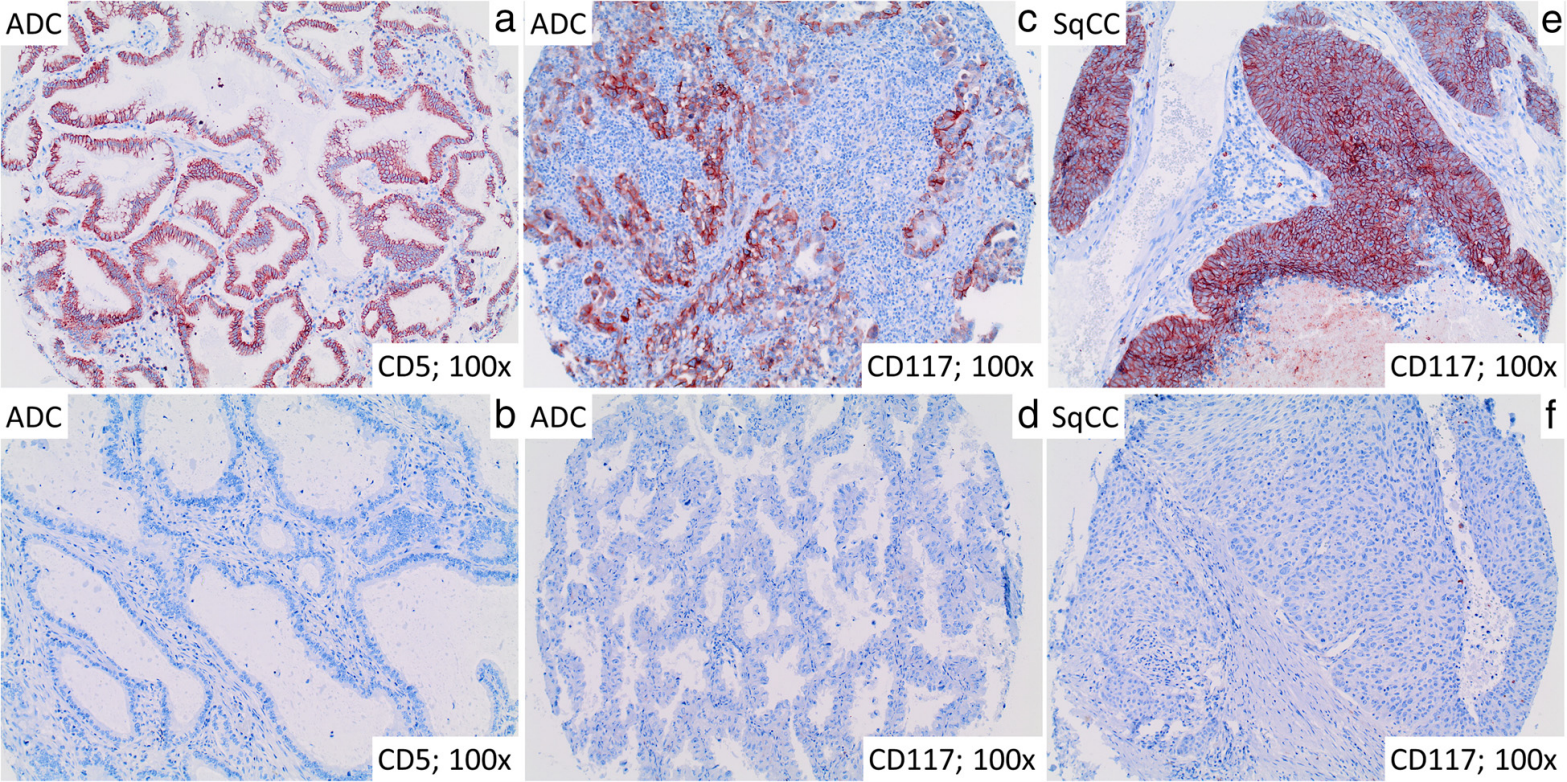
Representative immunohistological stainings of CD117 and CD5 in ADC and SqCC. Representative images of ADC of the lung show CD5 (**a**) and CD117 (**c**) positive and negative cases (**b**, **d**). Moreover, a CD117 (**e**) positive and CD117 (**f**) negative SqCC is illustrated

Both, CD117 and CD5 showed diffuse membranous and cytoplasmic positivity (Fig. 1). CD117 was positive in 145 out of 1457 evaluable cases (9.9 %) and CD5 was positive in 133 out of 1427 evaluable cases (9.3 %). 28 cases (1.9 %) showed coexpression of CD117 and CD5 (Table 1).

Among the 145 CD117 positive cases, 97 (66.8 %) were ADC, 34 (23.4 %) were SqCC, 5 (3.4 %) were ADSqCC, 8 (5.5 %) were LC, and one (0.6 %) was a PC.

The frequency of CD117 positive tumors was higher in ADC than in any other histotype (*p* < 0.01).

In the CD5 positive group consisting of 133 cases, 123 (92.5 %) were ADC, 0 (0 %) were SqCC, 4 (3.0 %) were ADSqCC, 3 (2.2 %) were LC and 3 (2.2 %) were PC. The frequency of CD5 positive tumors was higher in ADC than in any other histotype (*p* < 0.01). No SqCC showed expression of CD5.

**Fig. 2:**
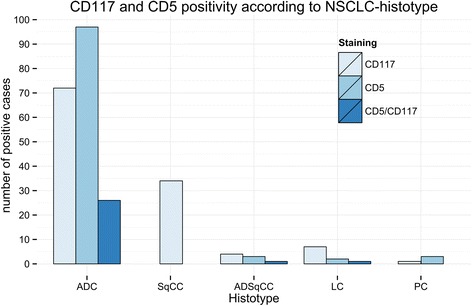
CD117 and CD5 positivity according to NSCLC histotype. A barplot of the absolute number of CD117- (lightblue), CD5- (blue) and CD117/CD5 (darkblue) positive NSCLC cases is shown according to the specific histotype. ADC: adenocarcinoma, SqCC: squamous cell carcinoma, ADSqCC: adeno-squamous carcinoma, LC: large cell carcinoma and PC: pleomorphic carcinoma

In the 28 cases that exhibited coexpression of CD117 and CD5, 26 were ADC (92.8 %), one was ADSqCC (3.5 %) and one was a LC (3.5 %). Again, CD5/CD117 coexpression was significantly more frequent in ADC than in any other histotype (*p* < 0.01). CD5 and CD5/CD117 positive patients were more often male (*p* < 0.01). Application of the immunoreactive score by Remmele et Stegner [16] could not improve the diagnostic power of any category. A summary of IHC findings is provided in Table 1 and Fig. 2.

## Discussion

This is the largest study analyzing the expression of CD5 and CD117 in NSCLC. We demonstrate that positivity for both markers is evident in approximately 10 % of all NSCLC and is predominantly found in ADC. While coexpression of CD5 and CD117 is rare and also predominates in ADC, the coexpression of both markers was not detected in SqCC. Likewise none of the SqCC showed CD5 expression. Thus, a marker panel including CD5 and CD117 is helpful in the differential diagnosis of primary lung and primary thymic squamous cell carcinomas with coexpression as a strong argument for a thymic primary.

Tumors of the mediastinum have a broad differential diagnosis ranging from benign disorders such as thyroid goiter or bronchogenic cysts to malignant diseases such as lymphoma, esophageal cancer or metastases from distant primaries [2]. Although routine thoracic imaging often initiate the subsequent evaluation of a mediastinal mass, it is rarely diagnostic and a biopsy is usually required to establish a definitive diagnosis. Albeit many entities have characteristic properties on H&E staining, tumor subtyping remains challenging in some cases and additional IHC stainings are necessary for a final classification. Specifically in the setting of central thoracic neoplasms the differentiation of thymic carcinomas from NSCLC with mediastinal involvement can be difficult [12, 17]. Immunostaining against CD117, a transmembrane tyrosine kinase receptor, and against CD5, a member of the ancient scavenger receptor superfamily, is routinely performed to separate thymoma from thymic carcinoma. While CD117 is positive in approximately 85 % of thymic carcinomas, CD5 is positive in approximately 70 %, and about 60 % of cases show a CD117 and CD5 positive phenotype [8–10]. Thus, positivity of both markers support a diagnosis of thymic carcinoma. While several studies have investigated the expression of both markers in thymic carcinomas [8–12, 18–20], knowledge on the prevalence in large scale NSCLC cohorts is limited. Hence, we questioned if these markers might also be helpful in the differential diagnosis to NSCLC.

Albeit most studies revealed CD117 positivity in 20 % [9, 18, 21] of NSCLC cases, a wide range varying from 7 % [22] to 64 % [23] has been reported, mainly due to different cut-offs applied. Since positive or negative is the most important diagnostic aspect and cut-offs are usually not helpful in the interpretation of diagnostic IHC markers in small biopsies, we used a dichotomous scoring scheme. Moreover, CD117 has been suggested to be a prognostic marker for NSCLC. However, data is conflicting since some studies [22–24] demonstrated a prognostic effect of CD117 positivity in NSCLC but others could not confirm this finding [25]. In the present study we found CD117 in 145 out of 1457 (9.9 %) NSCLC which matches the reported range. Most CD117 positive tumors were ADC (66.9 % of all CD117 positive cases), followed by SqCC (23.4 %) which is well in line with the available literature [18].

CD5 has been investigated in NSCLC before and found to be present in 0–85 % of cases [9, 10, 21]. CD5 positivity has been detected in a substantial subset of ADC in one study, but the results were limited by a rather small number of ADC investigated (*n* = 20) [21]. With regard to CD5 expression, a lack of representative large cohorts is also evident and the prevalence in NSCLC is unclear so far [9, 10, 21]. In this large scale study we found CD5 positivity in NSCLC in 133 out of 1427 NSCLC (9.2 %). Coexpression of CD117 and CD5 was detected in 28 out of 1465 cases (1.9 %) and was restricted to ADC, ADSqCC and LC. CD5 expression was not present in any of the 578 SqCC cases. Thus, CD5 or CD117/CD5 coexpression in a upper mediastinal tumor is a strong argument for a thymic primary and argues against SqCC of the lung.

## Conclusions

In summary, we demonstrate on 1465 NSCLC cases that a substantial subset exhibit CD117 and CD5 positivity and that pulmonary SqCC are consistently negative for CD5. Thus, the application of both immunomarkers is a valuable tool in the differential diagnosis of mediastinal tumors.

## Additional file

Additional file 1: Table S1. Antibodies applied in this study. (XLSX 10 kb)
